# Ankle patella: a report of a large accessory bone in the ankle: a case report

**DOI:** 10.4076/1757-1626-2-8512

**Published:** 2009-09-01

**Authors:** Yaghoub Salekzamani, Abolhassan Shakeri-Bavil, Nariman Nezami, Yousef Houshyar

**Affiliations:** 1 Department of Physical Medicine and Rehabilitation, Tabriz University (Medical Sciences), Imam Reza HospitalDaneshgah Street, TabrizIran; 2 Department of Radiology, Tabriz University (Medical Sciences), Imam Reza HospitalDaneshgah Street, TabrizIran; 3 Drug Applied Research Center, Tabriz University (Medical Sciences)Pashmineh, Daneshgah Street, TabrizIran; 4 Young Researchers Club, Tabriz Islamic Azad UniversityTabriz Islamic Azad University Complex, TabrizIran; 5 Department of Orthopedics, Tabriz University (Medical Sciences), Shohada HospitalGholshahr, TabrizIran

## Abstract

**Introduction:**

Sesamoids are ovoid bones with variable size and shape in the course of tendons, can be found in different parts of skeletal system.

**Case presentation:**

We report a case of 61-year-old woman in whom we observed a large accessory bone located in the anterior aspect of the left ankle joint. Since such accessory bones are found very infrequently, their presence may cause some diagnostic confusion.

**Conclusion:**

Regarding complaints in foot area one has to be familiar with such bones in order to make a correct diagnosis.

## Introduction

The term sesamoid coined by Galen is derived from the flat, oval seeds of Sesanum Indicum, an ancient East Indian plant used by Greek physicians as a purgative [1]. Sesamoid bones are usually ovoid nodules, a few millimeters in diameter but they vary in shape and size. Sesamoids can usually be found in the substance of tendon. The precise function of sesamoid bones is unclear but they may modify pressure, diminish friction and alter a tendon direction of pull and may also aid local circulation where a tendon is sharply deflected close to bone [2]. Sesamoids may be completely or partially ossified and the exact timing of ossification is unclear. Some sesamoids are described as constant such as the sesamoids of the first metatarsophalangeal joint but variable or accessory sesamoids are often described as accessory bones or ossicles. The patella, occurring in the tendon of the quadriceps femoris is the largest sesamoid bone in the body. In the foot and ankle, the accessory ossicles commonly observed are os tibiale externum, os trigonum and os perineum in order of frequency [3]. There are also some uncommon and rare sesamoids and accessory bones at the foot that are developmental anomalies that may occur as subdivisions of normal bones or as a separate prominence of an ordinary tarsal bone [4]. They may occur bilaterally or unilaterally. We report a case of a large accessory bone at the anterior of the ankle joint that may be the first observation of a large but extremely rare accessory bone.

## Case presentation

**Figure 1.**
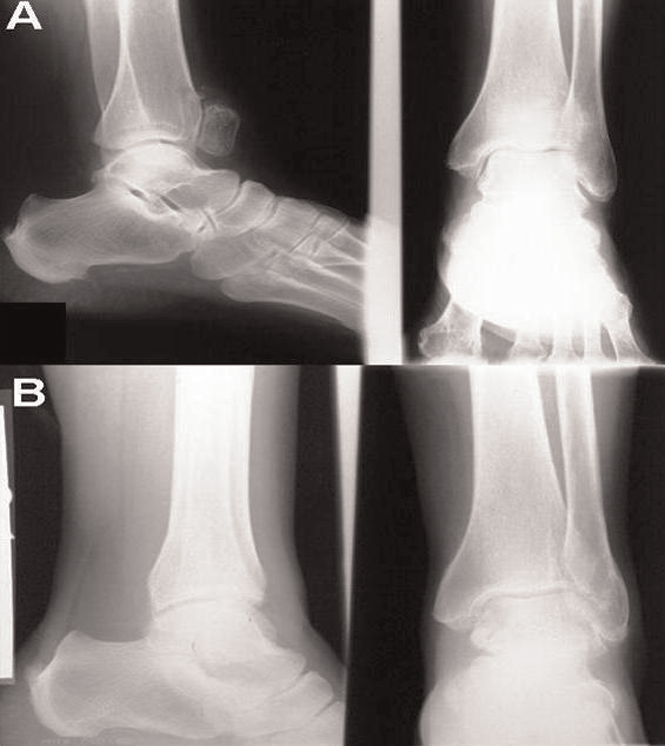
**(A)** Anteroposterior and lateral X-rays of left leg. **(B)** Anteroposterior and lateral X-rays of right leg.

**Figure 2.**
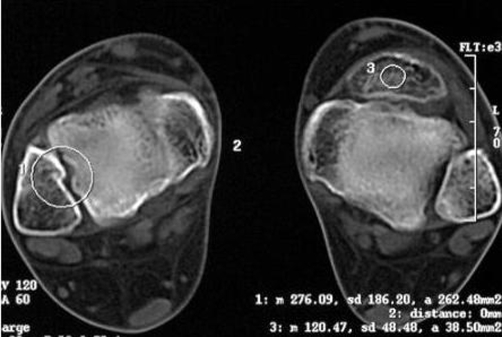
Computed tomography transverse plane of the left ankle was not shown any fracture or osseous pathology.

A 61-year-old woman, White Caucasian from Iranian origin, was referred to our outpatient rehabilitation clinic with pain in the left foot. Her main complaint was pain in the anterior side of left ankle joint mainly in heavy physical activity and in going upstairs. There was no history of trauma and her medical history revealed no abnormality. On examination, there was a stiff and immobile mass with 2-2.5 cm diameter located just anterior to the ankle joint. Patient stated that he have been had this protuberance since childhood. There was no swelling or tenderness. She described pain in dorsiflexion of the ankle joint. Plain radiographies showed the presence of a large accessory bone without osseous pathology (Figure 1A). X-ray evaluation of right foot and ankle were normal (Figure 1B). Computed tomography scans of the left ankle was not demonstrated any fracture or osseous pathology (Figure 2). All of evidences including history, physical examination and imaging study showed that accessory bone located in the tendon of tibialis anterior. Following diagnosis, conservative management with life style modification was advised for the patient.

## Discussion

Sesamoids are intertendinous bones and the most constant of them are the medial and lateral sesamoids of the first metatarsophalangeal joint. In contrast to them, accessory sesamoids are rare and occur elsewhere in the foot and ankle. Accessory bones are commonly considered as accidental findings and unrelated to the patient’s complaint [5]. In review of various reported accessory bones, there is not such a large accessory bone [3,6,7]. The radiographic appearance of this bone is like to the patella, so “Ankle Patella” could be a proper name for this accessory bone. Accessory bones rarely have clinical significance except for causing diagnostic confusion. A thorough knowledge of location of normal accessory bones is very important and localization of foot symptoms by history and physical examination are necessary to facilitate correct diagnosis and treatment and to avoid unnecessary invasive procedures.

There are several etiologies of sesamoid pain. The most commonly encountered are avascular necrosis, symptomatic bipartite sesamoid, fracture/trauma, osteomyelitis, or osteoarthritis [8]. Another possible etiology for sesamoid pain is arthritis of the sesamoid. Surgery is usually reserved until all conservative treatment has been exhausted and there is continued pain under the sesamoids.

Considering that the patient referred for first time and according to history, physical examination and imaging study which ruled out differential diagnosis of avascular necrosis, symptomatic bipartite sesamoid, fracture/trauma, osteomyelitis, osteoarthritis and arthritis, only conservative management was advised.
